# Phenotype-*loci* associations in networks of patients with rare disorders: application to assist in the diagnosis of novel clinical cases

**DOI:** 10.1038/s41431-018-0139-x

**Published:** 2018-06-26

**Authors:** Anibal Bueno, Rocío Rodríguez-López, Armando Reyes-Palomares, Elena Rojano, Manuel Corpas, Julián Nevado, Pablo Lapunzina, Francisca Sánchez-Jiménez, Juan A. G. Ranea

**Affiliations:** 1 Department of Molecular Biology and Biochemistry, University of Malaga, 29071 Malaga, Spain; 2 CIBER de Enfermedades Raras, ISCIII, Madrid, Spain; 3 European Molecular Biology Laboratory (EMBL) Heidelberg, Meyerhorfstrasse, 1, 69117 Heidelberg, Germany; 4 Future Business Centre, King’s Hedges Road, Cambridge, CB4 2HY UK; 5 Instituto de Genética Médica y Molecular (INGEMM), IdiPAZ, Hospital Universitario La Paz, Universidad Autónoma de Madrid, Madrid, Spain

## Abstract

Copy number variations (CNVs) are genomic structural variations (deletions, duplications, or translocations) that represent the 4.8–9.5% of human genome variation in healthy individuals. In some cases, CNVs can also lead to disease, being the etiology of many known rare genetic/genomic disorders. Despite the last advances in genomic sequencing and diagnosis, the pathological effects of many rare genetic variations remain unresolved, largely due to the low number of patients available for these cases, making it difficult to identify consistent patterns of genotype–phenotype relationships. We aimed to improve the identification of statistically consistent genotype–phenotype relationships by integrating all the genetic and clinical data of thousands of patients with rare genomic disorders (obtained from the DECIPHER database) into a phenotype–patient–genotype tripartite network. Then we assessed how our network approach could help in the characterization and diagnosis of novel cases in clinical genetics. The systematic approach implemented in this work is able to better define the relationships between phenotypes and specific *loci*, by exploiting large-scale association networks of phenotypes and genotypes in thousands of rare disease patients. The application of the described methodology facilitated the diagnosis of novel clinical cases, ranking phenotypes by *locus* specificity and reporting putative new clinical features that may suggest additional clinical follow-ups. In this work, the proof of concept developed over a set of novel clinical cases demonstrates that this network-based methodology might help improve the precision of patient clinical records and the characterization of rare syndromes.

## Introduction

Decades of advances in genomic technologies are increasing the accuracy in the field of genetic diagnosis. It is now widely accepted that deep phenotyping [1] and genotypic characterization of patients accelerates the identification of new genetic diseases and/or different disease subtypes with prognostic or therapeutic implications, as well as improves our understanding of human genetic diseases [2–4]. However, the accurate diagnosis of many genetic disorders becomes more complicated when patients show complex phenotypic profiles [5], when several genomic syndromes share clinical features among them, or when rare genetic aberrations affect an extremely low number of patients, as in rare diseases. Hence, key challenges for clinicians include the interpretation or classification of novel/extremely rare variants and the understanding of the phenotypic consequences of these genetic variations. A “genotype first” approach, in which patients are classified by a similar genomic rearrangement before a common clinical presentation is observed, has proven to be successful in characterizing the growing list of microdeletion/microduplication syndromes [6, 7].

The array-comparative genomic hybridization (aCGH) and single-nucleotide polymorphisms arrays (SNParrays), along with next-generation sequencing (NGS), are now the primary approaches used for copy number variation (CNV) detection [8]. CNVs are genomic structural variations that range from small variants (1 Kb) to larger structural changes (millions of nucleotides). These variations may correspond to deletions, duplications, or translocations found in genetic regions of individuals either inherited or by spontaneous occurrence (de novo), leading in some cases to disease [9]. CNVs are also present in healthy individuals, representing around the 4.8–9.5% of human genome variation in healthy individuals [8] of natural variation between genomes in the population. However, novel or inherited CNVs may be the cause of many disorders (as schizophrenia, Cron’s disease, or autism) and their identification and analysis are used for the diagnosis and characterization of many chromosomal syndromes [10–12]. In some laboratories, microarrays are a legacy technology that will be replaced by NGS. However, the still growing number of patients genotyped with aCGH/SNParrays platforms suggests a widespread usage of this technology. Indeed, public databases such as DECIPHER [4, 9, 13] show a significant amount of data originating from aCGH and SNParray technologies in recent years.

Nowadays, the complete identification of the phenotypic consequences of a given CNV remains challenging. Thus, it is imperative that new significant advances are achieved in the characterization of the genetic regions and molecular mechanisms controlling phenotypic expression.

To help with the characterization of molecular relationships between different phenotypes and microvariants, we aimed to apply principles of network medicine [14–18] to find the consequences of variants and their association with diseases. To this end, we focused on the development of a computational approach via tripartite networks made of three types of nodes: variants (CNVs), patients, and phenotypes. We used the DECIPHER database, a global repository of clinical patient data, as a resource for a systematic analysis and characterization of CNVs that are likely to affect function [4]. DECIPHER is a valuable resource that offers the phenotype and genotype records of a sizable number of patients with low prevalent genomic disorders, collected from more than 200 institutions from around the world [4, 9, 13]. Most patients with de novo CNVs in the DECIPHER database correspond to pediatric disorders related to developmental delay, mental retardation, or congenital structural anomalies [5, 19]. Along with CNVs, DECIPHER provides the pathological phenotypic profiles of the patients. This information is stored using a normalized vocabulary of phenotypes: the human phenotype ontology (HPO) [20], that facilitates the analysis and comparison between patient symptomatologies. In order to study the genotype–phenotype relationships in this dataset, we exploited the associations in our purposely built tripartite networks using the subset of patients presenting de novo CNVs in DECIPHER, identifying significant associations between mutated regions and pathological phenotypes. These phenotype-*locus* associations have been used to assess the potential of our network approach for assisting in the diagnosis of novel uncharacterized rare cases in clinical routine. This approach shows the potential of integrating information from thousands of characterized cases to identify novel genotype–phenotype patterns in rare and isolated cases with very scarce information to compare with.

## Materials and methods

### Source of datasets for building the networks

We used the de novo CNVs from DECIPHER patients with rare genomic disorders annotated and with available HPO terms (version 2014-05-08, mapped to the hg19 genome reference) through a Data Access Agreement with the database consortium. All phenotype and genotype data belong to patients that have provided informed consent to share their data in an anonymized way. These include the set of HPO terms annotated for each patient and their respective associated CNVs. The deletions subnetwork includes 2436 de novo CNVs from 2301 patients and 1795 HPO phenotypes. Duplications subnetwork is formed by 1114 de novo CNVs from 1013 patients, including 1160 HPO terms. DECIPHER selects the potential pathological CNVs, removing those observed in control populations. The DECIPHER 2014 dataset version was used to validate our hypothesis, because this version did not include data from the patients used here as novel clinical cases, as they were included in later versions (see next section), allowing us to test the feasibility of the network approach presented in this work. In our approach, we analyzed two types of relationships: (1) patients and genotypes (by CNVs) and (2) patients and phenotypes (by HPO terms). We subdivided CNVs into deletions and duplications, as we have previously observed that they may have different effects when affecting the same region (for example, in 19p13.3 and 19p13.13 microdeletion/microduplication syndromes [6, 21], microdeletions lead to macrocephaly while microduplications to microcephaly). In addition, we focused only on de novo variants, as they are the ones more likely to be associated with pathological phenotypes and the largest genomic rearrangements [22].

### Clinical case datasets used for testing our network approach

In order to test the feasibility of our network-based approach (for assisting in clinical diagnosis of patients with gains and deletions), two cohorts of patients provided by the INGEMM (Institute of Medical and Molecular Genetics, Hospital La Paz, Madrid, Spain) were used. The data obtained were in the same format as DECIPHER data: an anonymised set of CNVs and their correspondingly annotated HPO terms per patient. Clinical investigations were performed according to the guidelines of the Declaration of Helsinki [23], and patient’s data followed a strict ethical review process consisting of the signing of consent forms by patients (or their parents). We performed a proof of concept experiment of our technology in order to test if the associations found in the DECIPHER networks would help in the identification of the phenotypes associated to new pathological CNV cases from the INGEMM's sets of patients: (1) *Single clinical cases*: The first set of cases corresponds to a cohort of 293 patients (unpublished data) showing 519 genetic aberrations (312 deletions, 155 duplications, and 52 complex rearrangements), which were identified using oligonucleotide array CGH or SNParrays within 2010–2014 at the INGEMM. These patients were mainly referred to our clinics due to: intellectual disability, congenital malformations, and autistic spectrum disorder. (2) *A group of patients sharing phenotype and genotype, describing a new microdeletion/microduplication syndrome*: The second group of cases used was based on a specific syndrome characterization study, carried out by Nevado et al. [6], including 13 unrelated patients (with a total of 15 genomic rearrangements, distributed into 13 deletions and 2 duplications). Eleven of these patients had deletions and the remaining two duplications. The aCGH analysis together with clinical records showed that these patients shared phenotypic and genotypic features representing a novel interstitial microdeletion/microduplication syndrome [6]. Common features consist of: abnormal head circumference (macrocephaly for the deletions and microcephaly for the duplications), intellectual disability, developmental delay, hypotonia, speech delay, and some dysmorphic features.

#### Microarrays analyses

Array-CGH was performed using a custom oligonucleotide array (KaryoArray^®^ v3.0, 8 × 60K, Agilent-Based Technologies, Santa Clara, CA) [24]. Briefly, this array has an average density of one probe per 9 Kb in clinically relevant regions (microdeletion/microduplication syndromes, subtelomeric, and pericentromeric regions) and one probe per 175 Kb in other genomic regions. In some cases, a genome-wide scan of high density 850,000 tag SNParrays was conducted on probands, using the commercial design Illumina CytoSNP-850k BeadChip according to the manufacturer’s specifications (Illumina, San Diego, CA).

### Generating the network model

**Fig. 1:**
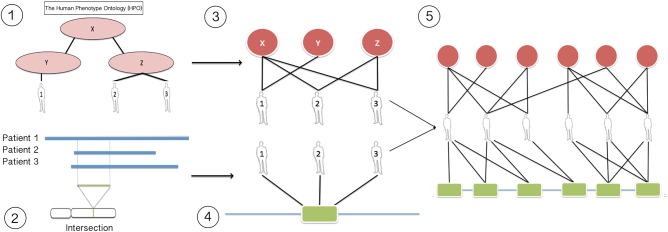
Generation of a tripartite network using DECIPHER patient data. Circles represent phenotypes and rectangles *loci*. (1) Patients are phenotypically annotated using HPO terms; (2) a *locus* is defined as the chromosomal region where a set of patient CNVs overlap; (3) the HPOs-patients layers; (4) the patients-*loci* layers; (5) the final tripartite network

Patient’s HPO terms and de novo CNVs associations data files were downloaded from the DECIPHER ftp server. As HPO is organized as a hierarchical tree (Fig. 1, panel 1), each patient was associated, in the network model, to his/her specific HPO terms (children) and all the parental terms above them in the HPO tree (Fig. 1, panel 3). A *locus* was defined as a SOR (small overlapping region) between patient CNVs used to build the network (Fig. 1, panel 2). A patient-*loci* network model (Fig. 1, panel 4) was generated connecting specific patients to *loci*. Panel 5 (Fig. 1) shows the integration of HPO phenotypes (red), *loci* (green), and patients. Thus, the layers of HPO terms and CNVs were connected by a middle layer of patients, forming a tripartite network, which was split in two halves; one for deletions and one for duplications. The deletions and duplications subnetworks generated 45,361 unique HPO term-patient/30,038 *loci*-patient associations and 17,010 unique HPO term-patient/10,888 *loci*-patient associations, respectively.

### Phenotype–genotype association measure calculation

**Fig. 2:**
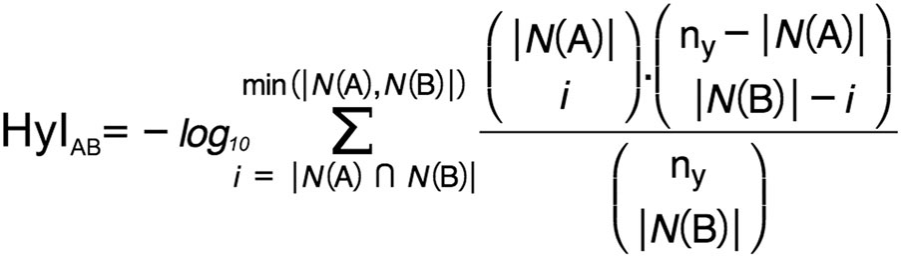
Hypergeometric Index (HyI) equation. 'A' represents a phenotype node and ‘B' a *locus* node within the tripartite network

**Fig. 3:**
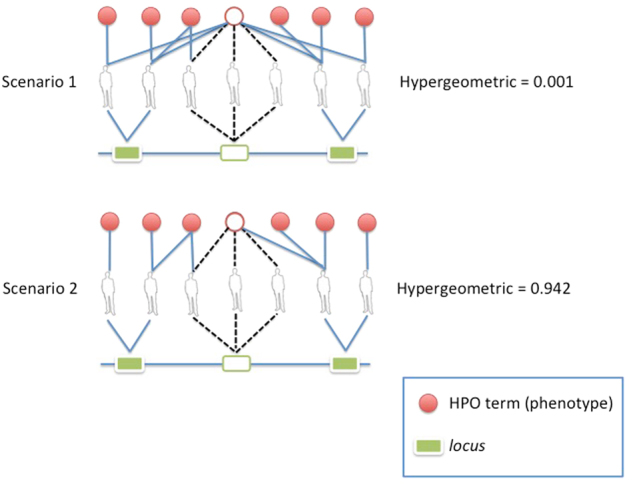
Calculation of the Hypergeometric Index (HyI) in two scenarios within a tripartite network. Scenarios 1 and 2 show the HyI values for high and low prevalent phenotypes, respectively, connected to a *locus* via three patients

We used the Hypergeometric Index (HyI) to measure the degree of association between HPO terms and *loci* through patient nodes in the tripartite networks (Fig. 2). The HyI yields the minus log-transformed probability of having an equal or greater level of interaction between a given phenotype-*locus* pair than the one expected by chance [25]. This index is frequently used to measure associations in different areas: e.g., microarray functional analysis, image pairwise comparison, data vectors, and spatial analysis in mass spectrometry [26–28]. We explained in detail the mathematical method, how it behaves and how to access the source code and instructions in Supplementary Material, Section 1. We also made a cross-validation of the method (Supplementary Material, Section 2), tested its possible dependencies (Supplementary Material, Section 3) and found (a) a negative relationship between HyI values and HPO frequency, (b) a positive correlation between HPO prevalence and the number of associated *loci*, and (c) a lack of correlation between HyI values and patients/CNVs per *locus*. And finally we show an example, analyzing a prevalent phenotype (Supplementary Material, Section 4). The significance of the association increases with the HyI value, since the lower the probability of the observed phenotype-*locus* association to be due to random the higher the HyI score value. This metric also dampens the effects of large CNVs overlapping with many different small CNVs, in two ways: (1) a widely spread phenotype usually leads to low HyI scores and (2) the phenotype will be highly rated only if it is shared by a big number of patients in the same locus (SOR). Both facts, related to specificity, are discussed in Supplementary Material. An example of how HyI works is shown in Fig. 3, with two putative scenarios. Scenario 1 in Fig. 3 shows a phenotype connected to a *locus* via three different patients, but this phenotype has four more connections to other patients with genomic disorders located in different *loci* (high prevalent HPO phenotype). The HyI association value obtained in this case is low (HyI = 0.001; *p*-value = 0.99). In Scenario 2, a phenotype is also connected to a *locus* by three patients, but in this case the amount of connections to other patients and *loci* for that phenotype is low; this latter case represents a more specific phenotype-*locus* association, and therefore the significance is higher (HyI = 0.942; *p*-value = 0.11). In order to establish a significance threshold, for this study, we considered HyI value ≥2.0 as significant HPO phenotype-*loci* associations (a *p*-value ≤ 0.01 due to random).

### Measuring the association index between HPO phenotypes and *loci* in the whole DECIPHER network

To calculate the significance of associations between HPO phenotypes and *loci*, we applied the hypergeometric association index (HyI) to the de novo deletions and de novo duplications tripartite subnetworks (see section above). We calculated the HyI association score for 600,234 different HPO term-*locus* pairs using the deletions subnetwork, and 175,956 using the duplications subnetwork. Some examples of HPO term-*locus* associations are shown in Table 1 and the rest of them are included in the Supplementary Material that accompanies this work.

**Table 1:** Examples of HPO-phenotypes vs. *locus* associations identified in the system

HPO code	Phenotype	Max HyI (del.)	Max HyI (dup.)	*Locus* coordinates (del.)	*Locus* coordinates (dup.)
HP:0002813	Abnormality of limb bone morphology	4.93	3.10	Ch 7: 41613503–42807486	Ch 9: 11818351–12709928
HP:0000284	Abnormality of ocular region	3.39	1.65	Ch 7: 41518389–41613502	Ch Y: 945080–2654860
HP:0000153	Abnormality of the mouth	3.50	1.94	Ch 2: 200208169–200246437	Ch 22: 40849826–41082043
HP:0001315	Reduced tendon reflexes	3.16	3.54	Ch 3: 6036656–6045520	Ch 20: 29462074–29833608
HP:0010477	Aplasia of the bladder	–	3.36	–	Ch 17: 34817222–34817420
HP:0001933	Subcutaneous hemorrhage	3.38	–	Ch 21: 15398168–15412670	–
HP:0200008	Intestinal polyposis	3.56	–	Ch 10: 89717525–93614902	–
HP:0001789	Hydrops fetalis	3.56	–	Ch 13: 80378611–80386671	–
HP:0003186	Inverted nipples	2.90	3.54	Ch X: 455566–544731	Ch 16: 75683739–78186860
HP:0000699	Diastema	3.08	3.36	Ch 5: 13750113–14064732	Ch 7: 2290686–2996437
HP:0010761	Broad columella	3.56	–	Ch 19: 48066340–48270667	–
HP:0008110	Equinovarus deformity	3.26	3.36	Ch 16: 2038810–2124458	Ch 16: 90148342–90148393
HP:0002323	Anencephaly	–	3.36	–	Ch 17: 34817222–34817420

Columns: (1) HPO code; (2) Phenotype description; (3) Maximun HyI obtained for the phenotype associated to a locus in the de novo deletions subnetwork; (4) Maximun HyI obtained for the phenotype associated to a locus in the de novo duplications subnetwork; (5) Chromosome id: the start and end coordinates (in bps in the hg19 genome reference) of the locus associated with the phenotype with the Max HyI value in the de novo deletions subnetwork; and (6) in the de novo duplications subnetwork

### Ranking putative phenotype/CNV associations in novel uncharacterized clinical cases

**Fig. 4:**
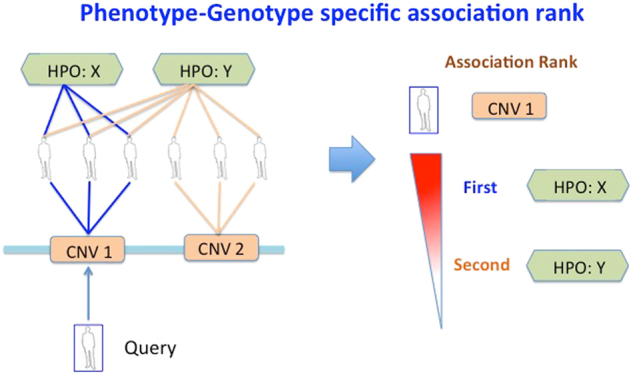
Identification of phenotype-*locus* associations for new clinical cases. A CNV from a new patient (Query) is assigned to a *locus* (CNV 1) in the tripartite network by genomic overlap comparison (left side of the figure). All the phenotypes associated to patients are ranked based on their HyI association value to the query *locus* (right side)

We calculated HyI values for the de novo deletions and de novo duplications subnetworks. A dataset was set up to include all the HyI scores for all HPO phenotypes against all *loci*. We also used this dataset to identify phenotype–genotype associations, ranked by their HyI value, for new patients with CNVs that were not included in the DECIPHER dataset (Fig. 4).

### Other parameters used

In addition to HyI association value, we also defined and calculated some additional parameters in order to estimate more accurately the significance of our results: *Penetrance*: We defined penetrance as the percentage of patients in the DECIPHER database with the same affected *locus* sharing the same HPO term. A penetrance of 100% means that all patients having affected the *locus* express the phenotype. Penetrance is a useful parameter for measuring the effects of combined genetic aberrations and the probability of showing the associated phenotype if a patient harbors a variant in that specific region. *% max*: The ratio between the HyI value obtained for an HPO term associated to a *locus* and the maximum HyI value in the network for that phenotype. A *% max* of 100% means that the HPO phenotype-*locus* HyI value is the maximum found for that HPO phenotype in the whole genome. *Locus overlap*: The percentage of overlap (in base pairs) a CNV from a novel clinical case has with a *locus* present in our reference network. If this parameter is 100%, that means that the “case” CNV is contained in the *locus* region. *Locus* overlap has to be taken into account in the interpretation of the data, since if the query patient just shared a tiny percentage with the studied *locus*, it may not affect the specific region responsible for a particular phenotype associated to that *locus* (even if the HyI value between the *locus* and the phenotype is high).

## Results

### Application of the networks analyses to novel clinical cases

We used herein 293 clinical cases (patients), associated with 519 CNVs, diagnosed at the INGEMM (Institute of Medical and Molecular Genetics, Hospital La Paz, Madrid, Spain) during the period 2010–2014. For 258 out of the 293 clinical cases (88%) our approach found at least an overlapping CNV with a pathological *locus*, and for each pathological *locus* a list of HPO phenotypes sorted by their HyI value was provided (as described in Methods, section “Ranking putative phenotype/CNV associations in novel uncharacterized clinical cases”, Fig. 4). The resulting ranked list for each pathological *locus* only included HPO terms with a HyI value ≥2.0 (*p*-value ≤ 0.01). 17,096 significant (HyI ≥ 2.0) associations were found involving 856 different phenotypes. A total of 381 out of the 1489 HPO terms (26%) diagnosed by clinicians were also identified by our system associated to a patient’s CNV in the de novo deletions subnetwork, and 252 out of 609 (41%) in the de novo duplications subnetwork (Table 2). On the other hand, a total of 521 and 376 non-diagnosed HPO phenotypes, for deletion and duplication respectively, were identified by our method to be associated with disorders in the clinical cases (Table 2). These results indicate that this novel approach could be extensively used for differential diagnosis of novel clinical cases in order to find those phenotypes associated with single CNVs through comparison to the entire patient information integrated in the network generated from DECIPHER.

**Table 2:** Statistics of the comparison between the clinical records of the 293 patients with rare CNV genomic disorders and the HPO phenotypes-loci associations identified by the system

# HPO phenotypes diagnosed by the INGEMM clinicians for all the patients	1694
# Diagnosed HPOs for all the patients presenting a deletion	1489
# Diagnosed HPOs for all the patients presenting a duplication	609
# Diagnosed HPOs also identified by the system using the de novo deletions network	381
# Diagnosed HPOs also identified by the system using the de novo duplication network	252
# HPOs identified by the system and not diagnosed (with HyI > 2, penetrance 100%, and loci overlap 100%) using the de novo deletions network	521
# HPOs identified by the system and not diagnosed (with HyI > 2, penetrance 100%, and loci overlap 100%) using the de novo duplications network	376

In order to illustrate the potential utility of this methodology for assisting in a genetic diagnosis, we select and discuss in detail three clinical cases (two deletions and one duplication) from our cohort (Table 3). Interestingly, patients presented as examples in Table 3 show a high level of similarity matching between phenotypes diagnosed by clinicians and those identified by our network association system. However, in the first case the system finds a couple of phenotypes: “Macrocephaly” and “Abnormality of joint mobility” (highlighted in dark in Table 3), which were not reported originally by the clinicians. Although, *penetrance* and *% max* parameters are relatively low for both phenotypes, our results suggest the need for carrying out some additional clinical tests to confirm or discard these phenotypes in the patient.

**Table 3:** Example of the CNV analysis for three INGEMM´s patients

Patient 1	Deletion	chr 12	Mutation start hg19: 30 946 782		Mutation end hg19: 132 246 215
**Observed phenotypes**	**Associated phenotypes**	**HyI rank**	**Penetrance**	**% max**	**Node overlap**
Abnormal facial shape (HP:0001999)	+ Low-set ears (HP:0000369)	5.41	100%	100%	100%
	-> Abnormal location of ears (HP:0000357)	5.16	100%	100%	100%
	-> Abnormality of the outer ear (HP:0000356)	4.46	100%	100%	100%
	-> Abnormality of the ear (HP:0000598)	4.01	100%	100%	100%
*Not reported*	*+ Macrocephaly (HP:0000256)*	*3.63*	*75%*	*100%*	*100%*
	*+ Abnormality of joint mobility (HP:0011729)*	*3.34*	*75%*	*85%*	*100%*
	*-> Abnormal joint morphology (HP:0001367)*	*3.09*	*75%*	*63%*	*100%*
Global developmental delay (HP:0001263)	+ Abnormality of body weight (HP:0004323)	3.28	100%	100%	100%
	-> Growth abnormality (HP:0001507)	2.68	100%	67%	100%
	+ Short stature (HP:0004322)	3.03	100%	70%	100%
	-> Abnormality of body height (HP:0000002)	2.70	100%	72%	100%
	-> Growth delay (HP:0001510)	2.63	100%	50%	100%

Patient 2	Deletion	chr 17	Mutation start hg19: 34 911 952		Mutation end hg19: 36 510 799
**Observed phenotypes**	**Associated phenotypes**	**HyI rank**	**Penetrance**	**% max**	**Node overlap**
Multicystic kidney dysplasia (HP:0000003)	+ Abnormality of the kidney (HP:0000077)	3.18	50%	71%	100%
	-> Abnormality of the genitourinary system (HP:0000119)	2.87	67%	72%	100%
	-> Abnormality of the upper urinary tract (HP:0010935)	2.94	50%	70%	100%
	-> Abnormality of the urinary system (HP:0000079)	2.45	50%	66%	100%
*Fetal choroid plexus cysts (HP:0011426)*	*Not found*	*Not found*	*Not found*	*Not found*	*Not found*

Patient 3	Duplication	chr 16	Mutation start hg19: 22 369 809		Mutation end hg19: 22 436 522
**Observed phenotypes**	**Associated phenotypes**	**HyI rank**	**Penetrance**	**% max**	**Node overlap**
Abnormal facial shape (HP:0001999)	+ Depressed nasal bridge (HP:0005280)	3.45	100%	100%	100%
	+ Deviated nasal septum (HP:0004411)	3.04	33%	100%	100%
	-> Abnormality of the nasal bridge (HP:0000422)	2.48	100%	100%	100%
	-> Abnormality of the nose (HP:0000366)	2.21	75%	80%	100%
	-> Abnormality of the midface (HP:0000309)	2.37	50%	89%	100%
	+ Malar flattening (HP:0000272)	3.14	50%	91%	100%
	-> Abnormality of the zygomatic arch (HP:0005557)	3.13	50%	91%	100%
	Brittle hair (HP:0002299)	2.82	50%	100%	28%
*Global developmental delay (HP:0001263)*	*Not found*	*Not found*	*Not found*	*Not found*	*Not found*

Header: Chromosome coordinates of the CNV (mapped to the hg19 genome reference). Columns from left to right: (1) Observed phenotypes in the patient reported by the physician; (2) Associated phenotypes: The list of phenotypes identified in the network with a significant level of association (HyI value) with the overlapping locus to the patient CNV. “+” indicates that the HPO term is the most specific one among a group of them (children). “>” is used for detected parental terms that are related to the child term (less specific terms) in the HPO ontology; (3) HyI (Hypergeometric Index) Rank; (4) Penetrance; (5) % max; (6) Node overlap.

The CNV of patient 2 has a region of about 100 Kb that does not match any *locus* (non-matching region coordinates -hg19-: 36 410 558–36 510 799 bps in chr17). In this case, patient 2 was diagnosed with “Fetal choroid plexus cysts”, an anomaly consisting of small fluid-filled structures within the choroid of the lateral ventricles of the fetal brain. These results suggest that the non-matching CNV region from the clinical case could harbor the genetic cause of the “Fetal choroid plexus cysts” diagnosed in this patient. This shows a potential use for the network approach in discriminating the particular regions associated with different phenotypes.

Finally, we show an example of a patient with a duplication (patient 3 in Table 3) where the clinicians observed two phenotypes: “Abnormal facial shape” and “Global developmental delay”. The second phenotype was not detected by our system, but for the first one we detected up to eight ontologically related phenotypes with a significant HyI association value. The most specific phenotypes were the following: “Depressed nasal bridge”, “Deviated nasal septum”, “Malar flattening”, and “Brittle hair”. Just to note that, although “Brittle hair” has a significant HyI (2.82) it shows low values for *penetrance* and *node overlap*, so this should be carefully considered. Taking into account this information, we can infer that the “Abnormal facial shape” observed in this patient may be related to nose malformations and the structure of the zygomatic arch.

### Application of the methodology to a set of patients who share a novel non-recurrent microdeletion/microduplication syndrome

The same approach, described in the section above for single clinical cases, was also applied to a set of 13 patients with 15 CNVs (13 deletions and 2 duplications), previously classified by reverse genetics in the INGEMM clinical unit into a new microdeletion/microduplication syndrome located at the 19p13.3 genomic region [6]. All these patients share a number of phenotypes related to a similar CNV rearrangement (deletions and duplications). The summary of the comparison between phenotypes established by Nevado et al. [6] and the results of our analysis are shown in Table 4. These results show that our systematic approach was able to identify 37 out of the 178 diagnosed phenotype-patient associations for this syndrome (21%) with significant HyI values (HyI ≥ 2.0; *p*-value ≤ 0.01). Although we recommend the use of HyI values above 2.0 to obtain highly reliable results, our system also provides, as additional information, results with lower values, which must be taken with caution and be evaluated for each particular case, considering additional information in order to make any inference. In this sense, 91 out of the 178 diagnosed phenotype-patient associations for this syndrome (51%) were also detected by our system but with HyI < 2.0. Some of these cases correspond to prevalent phenotypes, such as “psychomotor development delay” or “intellectual disability”. We differentiate both types of results by using different colors in the grids of Table 4. There is a set of phenotypes diagnosed in most patients with this syndrome that were also recurrently found by our systematic approach, with significant HyI values. For example: “Wide nasal bridge” (9 associations found by the system out of 10 patients diagnosed), “Gastro-esophageal reflux” (4 out of 4 diagnosed), “Umbilical hernias” (4 out of 4), “Heart disease” (5 out of 7), and “Feeding problems” (5 out of 6). As we showed previously, our system also found phenotypes associated with these CNVs in 46% of the patients with this syndrome that have not been reported in the patients’ clinical records, such as: “abnormality of the kidney”, “abnormality of the penis”, and “abnormality of connective tissue”. In a retrospective review (carried out after the application of our method to check our predictions directly in patients) of 38 of these patients with 19p13.3 microdeletions, renal anomalies were found in 26.31% of them, anomalies of the sexual organs in 21.05%, and there were no known cases of abnormality of the connective tissue. These results support the potential of the system to assist clinical diagnosis (see Section 5, Supplementary Material).

**Table 4:** Patients with the syndrome associated with CNVs in the 19p13.3 region

The table shows the patient ids; Type of CNV: “−” for deletions and “+” for duplications. First column: phenotype descriptions and HPO codes. Boxes with X indicate that the phenotype has been previously diagnosed in the corresponding patient by the clinician examination. Phenotypes found by the systemic approach with significant HyI values (HyI value ≥ 2.0; *p*-value ≤ 0.01) are represented by dark gray boxes, and those detected with lower HyI values by light gray boxes. Phenotypes are grouped in four general categories: Growth and development, neurology, others, and an extra category for those found by the system but not previously diagnosed

## Discussion

We developed and showed herein a new approach to assess relationships between genotypes (using CNVs) and phenotypes (using HPO terms) in order to help in the diagnosis of rare genomic syndromes. We used the HPO [2], which provides a structured, comprehensive, and well-defined set of over 11,000 terms that characterize phenotypic abnormalities seen in human disease [20]. Many algorithms and computational tools use the HPO extensively. In fact, it is useful for clinical differential diagnostics, as well as for the prioritization of candidate disease-associated genes in exome sequencing studies [29]. As an example, the web application Phenomizer [30], which analyzes relationships between human phenotypic abnormalities and diseases, makes use of HPO database.

The systematic mathematical approach implemented in this work is able to establish a fine-tuning of the relationships between phenotypes and specific *loci* by exploiting large-scale association networks of phenotypes and genotypes in thousands of patients with rare disorders and complex pathologies. Clinicians can directly associate the variants of the patients and their phenotypes when they co-occur in the same *locus*, but they cannot easily differentiate the specificity degree and the association statistical significance of each phenotype associated to each particular *locus*, as the system implemented in this work does. Our results clearly support the use of this tool to identify potential *loci* involved in genetic diseases within a database such as DECIPHER, whose approximately half of its patients are currently not associated to genetic syndromic entities. The application of the described methodology in a set of novel clinical cases, used as proof of concept, has shown a high potential to facilitate the diagnosis of these novel unsolved clinical cases, ranking phenotypes by *locus* specificity and reporting putative new phenotypes. Indeed, these phenotypes may suggest additional clinical explorations that could help improve the precision of patient diagnosis and the characterization of new rare syndromes, as evidenced. The results obtained here indicate that the comparative analysis of new clinical cases with variant-phenotype associations identified in the network of formerly diagnosed patients could have important applications in the design of customized arrays and NGS approaches for genetic-variant diagnosis, as well as in the search of candidate genes associated to the different mutated genomic regions observed in patients.

Currently available methodology for genetic diagnosis include PhenIX [31], Phenomantics [32], eXtasy [33], PHIVE, hiPHIVE [34, 35], Phevor [36], Phen-Gen [37], and OMIM-Explorer [29]. These tools provide phenotypically supported annotations to particular variants detected in patients, yielding rankings for pathological variants or genes. Some of these methods (PhenIX, PHIVE, hiPHIVE, or OMIM-Explorer) may provide, directly or indirectly, a guide for disease diagnosis. Particularly useful is Phenomizer, which compares diseases and patient phenotypes using the Online Mendelian Inheritance in Man (OMIM) disease catalog [30]. However, OMIM-Explorer is the only tool that provides phenotype suggestion for differential presumptive diagnosis, as the system presented in this work also does. The main difference between OMIM-Explorer and our network-approach is that the former uses available annotations of: genes, variants, diseases, and phenotypes in OMIM and other similar databases, while in this work the variants-phenotypes associations are directly inferred from a network of individual patients. In addition, another important difference in our tool is that it manages a tripartite (variants–patients–phenotypes) network made of pathological de novo CNVs present in patients with rare genomic disorders from the DECIPHER database. This characteristic makes the DECIPHER patients network approach presented here especially suitable for the comparative diagnosis of rare and orphan diseases with a genetic origin (80% of all rare diseases), a clinical field that is specially challenging due to the low number of patients and the lack of computational methods for helping diagnosis [38–40].

## Electronic supplementary material

Supplementary Material
