# Supplementary material for: Phenotype-loci associations in networks of patients with rare disorders: application to assist in the diagnosis of novel clinical cases
Source: Eur J Hum Genet. 2018 Jun 26;26(10):1451–61. doi: 10.1038/s41431-018-0139-x (PMC6138686; doi:10.1038/s41431-018-0139-x)
Supplement: Supplementary file 1 — Supplementary Material [file 41431_2018_139_MOESM1_ESM.pdf]

SECTION 1. Descriptions of the different metrics applied for the analysis of the genotypes-patients-phenotypes network

**Table S1. Studied Network Analysis Algorithms**	
**Jaccard Index**	Similarity index based on the proportion between shared nodes (for A and B) and the total number of nodes connected to A or B.
$$J_{AB} = \frac{\lvert N(A) \cap N(B) \rvert}{\lvert N(A) \cup N(B) \rvert}$$	
**Pearson Correlation Coefficient Index**	Correlation between interaction profiles of A and B.
$$PCC_{AB} = \frac{\lvert N(A) \cap N(B) \rvert \cdot n_y - \lvert N(A) \rvert \cdot \lvert N(B) \rvert}{\sqrt{\lvert N(A) \rvert \cdot \lvert N(B) \rvert \cdot \left(n_y - \lvert N(A) \rvert\right) \cdot \left(n_y - \lvert N(B) \rvert\right)}}$$	
**Hypergeometric Index**	Logarithmic transformation of the probability of obtaining an overlap in the interaction greater or equal than the observed between A and B.<br><br>Where:<br><br>A and B are nodes of a different nature connected by an intermediate layer (CNVs and Phenotypes in our study).<br><br>The degree of a node X (|N(X)|) is defined as the number of nodes with which it interacts.<br><br>(|N(A) ∩ N(B)|) represents the shared partners between A and B.<br><br>(n_y) is the total number of nodes in the intermediate layer (patients).
$$HyI_{AB} = -\log \sum_{i=\lvert N(A) \cap N(B) \rvert}^{\min(\lvert N(A), N(B) \rvert)} \frac{\binom{\lvert N(A) \rvert}{i} \cdot \binom{n_y - \lvert N(A) \rvert}{\lvert N(B) \rvert - i}}{\binom{n_y}{\lvert N(B) \rvert}}$$	

We show in table S1 the main association indices we tested in this work. The Jaccard metric is the simplest one, it is algorithmically light to compute and easy to interpret, but it returns a rudimentary spectrum of solutions with a low level of accuracy, considering the proportion of shared nodes but without taking into account the total number of nodes in the network. However, the Pearson Correlation Coefficient (PCC)

measures the linear relationship between two interaction profiles considering if the interactions are present or absent, returning a value between 1 (perfect correlation of profiles) and -1 (perfect anticorrelation), being 0 associated to a random comparison. In biological networks, shared non-partners can be as essential as shared ones. Finally, the Hypergeometric test needs a higher level of computational resources but it provides fine-tuning results, and what is even more important, it measures the statistical significance rather than the raw magnitude, determining the likelihood of observing a certain overlap between the interaction profiles of two given nodes. This constitutes the most powerful metric for our analysis. In Figure S1 we show the behaviors of these three metrics in different situations.

We have made available the main scripts of the methodology developed in this work and the documentation with the instructions to use it. The scripts and guides are available at: https://github.com/bio267lab/HyI

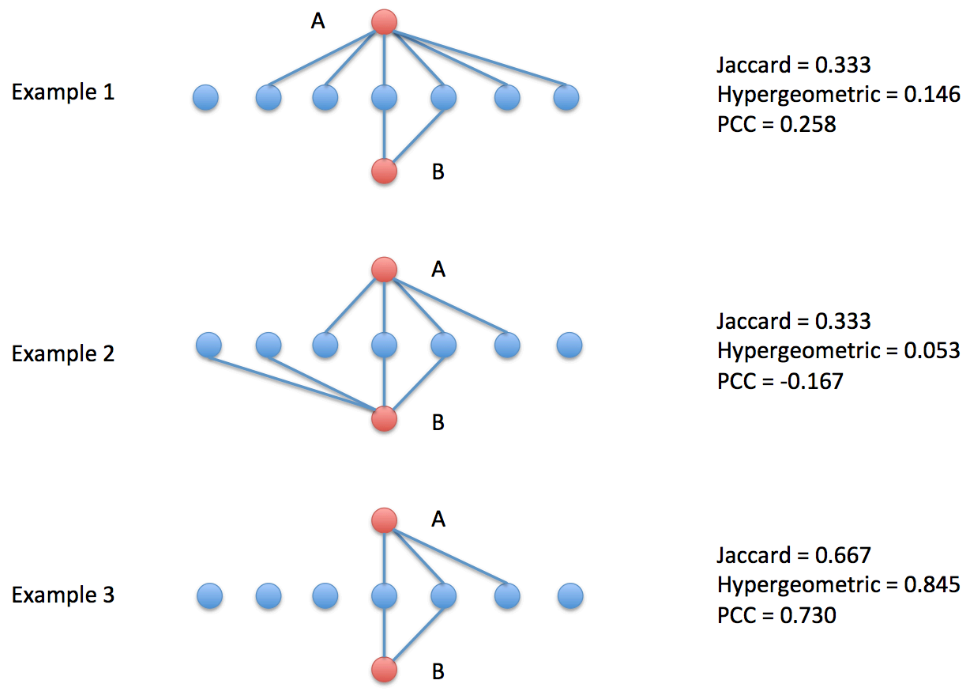

***Figure S1**. Metrics behavior examples: Jaccard, PCC and Hypergeometric measures give the best score to the third example among the three, but they have some differences. The Jaccard metric is unable to distinguish between example 1 and example 2 despite both examples being quite different in terms of similarity of profiles between A and B. Meanwhile, PCC penalizes the second example due to the fact that 50% of A and B connections are not shared, and the metric detects an anticorrelation. The Hypergeometric Index, on the other hand, returns values between 0 and 1, scoring the lowest value for example 2 and the highest for example 3, showing a great level of discrimination.*

We would expect these metrics to behave in a reasonable similar way, since they are all measuring network connectivity and profile similarity. In order to check that assumption, we performed a set of comparative plots between the results of the different

methods (see Fig. S2 and Section 3). The results showed that although there is a correlation between metrics, HyI shows the best performance. Finally, and taking into account that correlation, the validation benchmarking and the intrinsic nature of each metric, we decided to use the Hypergeometric index in order to build the genotype-phenotype association method, for being more accurate and include an intrinsic statistical significance value.

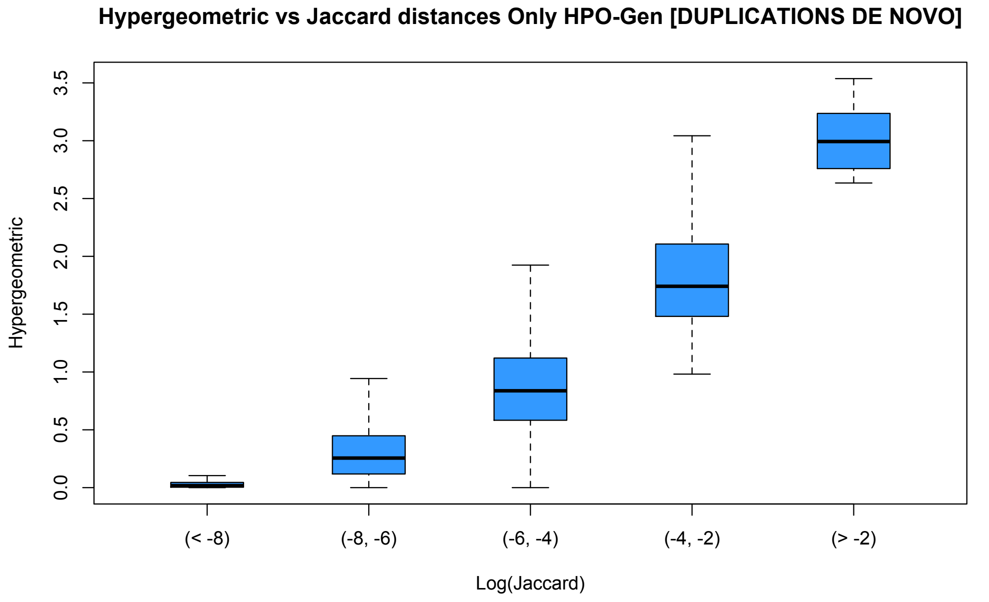

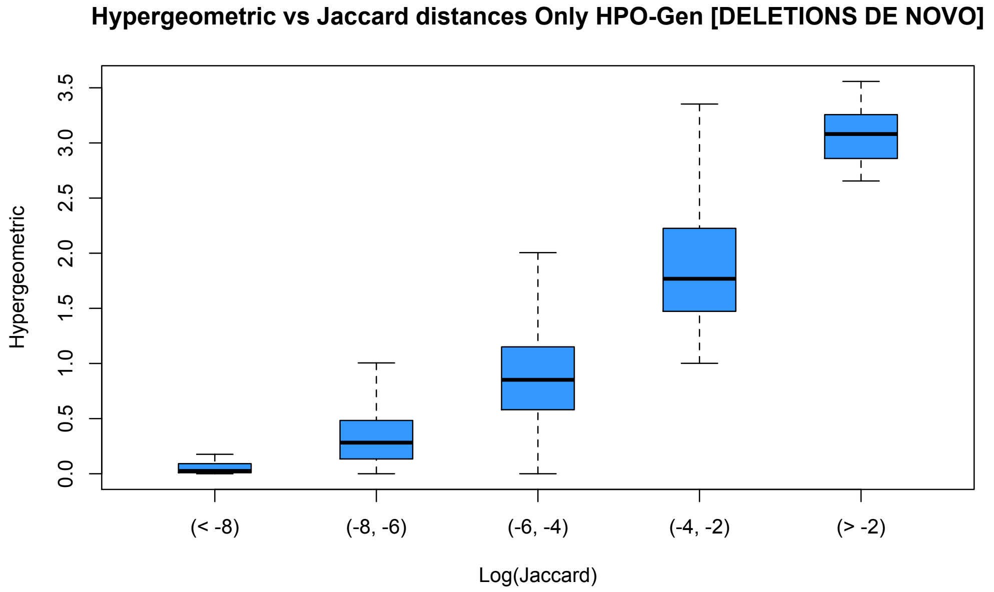

Figure S2. *Comparative of HyI (Hypergeometric) and Jaccard metrics distributions for the same DECIPHER tripartite networks of duplications de novo (upper plot) and deletions de novo (lower plot).*

<u>**SECTION 2. Validation and benchmarking of the association metrics.**</u>

For validating our method, we performed a 10-fold cross-validation over our data, randomly splitting the DECIPHER patients dataset into 10 sub-samples: each one of these sub-samples was iteratively used as positive dataset of phenotype-CNV associations and the remaining 90% of patients were used to build the tripartite network (training set).

The phenotypes-*loci* association values were calculated on every tripartite network using three methods: Hypergeometric Index (HyI), Pearson Correlation Coefficient (PCC) and Jaccard; and the precision/recall curve, for each association measure, was calculated using the methodology described in Pandey *et al*. 2007, and plotted together (see Fig S3).

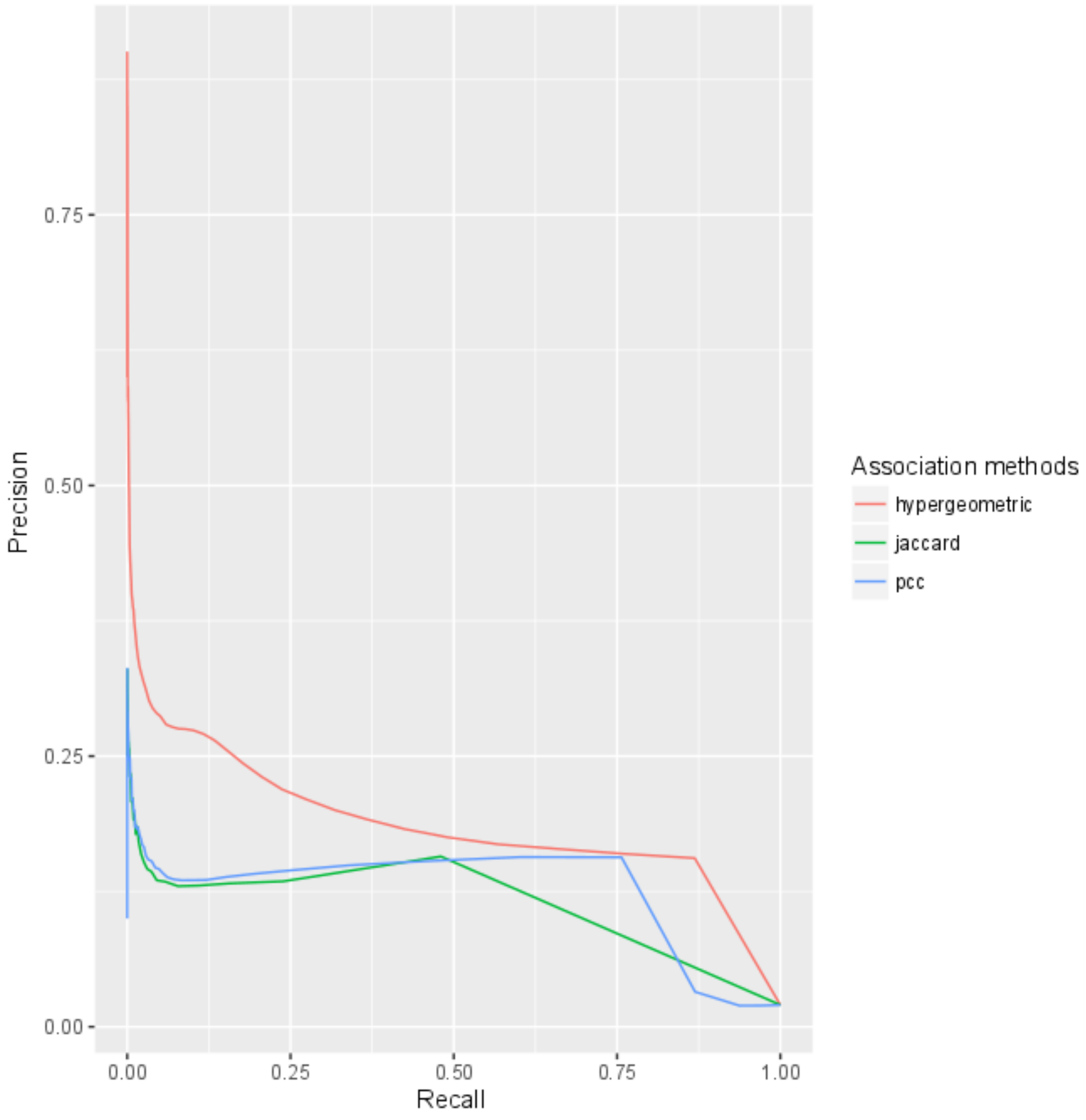

Figure S3. *Precision vs. recall curve for the HyI (red), PCC (blue) and Jaccard (green) association indices using DECIPHER data. Precision (prec) and recall (rec) values are set from 0 to 1.*

Benchmarking the comparison (precision vs recall curves) amongst the three indices shows a significantly better performance of the HyI compared to Jaccard and PCC measures. These results support the use of the HyI to measure the phenotype-*loci* associations in this work.

References:

Pandey, G., Steinbach, M., Gupta, R., Garg, T. & Kumar, V. Association analysis-based transformations for protein interaction networks: A function prediction case study. *Proc. ACM SIGKDD Int. Conf. Knowl. Discov. Data Min.* 540–549 (2007). doi:10.1145/1281192.1281251.

SECTION 3. Comparative statistics amongst the Hypergeometric Index (HyI) values, HPO frequencies, and the number of patients/CNVs.

In order to study possible dependencies we carried out different analyses comparing the relationship between the HPO phenotypes and the patients/CNVs frequencies in the whole network and their relationships with the HyI values distribution.

Phenotype prevalence, associated *loci* and HyI values.

The distribution plot that represents HPO frequency vs. HyI values (Fig. S4) shows a negative relationship: the higher the frequency of HPO terms the lower the distribution range of HyI values. Additionally, it is observed a positive relationship between phenotype prevalence (HPO frequency) and the number of associated *loci* when these two variables are plotted (Fig. S5). This negative relationship with HyI values and the positive correlation with the number of *loci* indicates that high prevalent phenotypes in the DECIPHER patients dataset tend to be associated to more *loci* than low prevalent ones, increasing the probability of being associated to a *locus* by chance (HyI null hypothesis). This consequently reduces the general HyI values of phenotypes-*loci* associations as it is illustrated in the scenario 1 in Figure 3 of the main manuscript.

Conversely, less frequent phenotypes show distributions with wider and higher range of HyI values compared to more prevalent ones (e.g. phenotype frequency < 0.001, see Fig. S4). These results suggest that low prevalent phenotypes have less probability than higher prevalent ones to be associated by chance (HyI null hypothesis) to the same *locus*, allowing us to identify more significant (high HyI values) phenotype-*locus* associations, as it is illustrated in the scenario 2 in Figure 3 of the main manuscript.

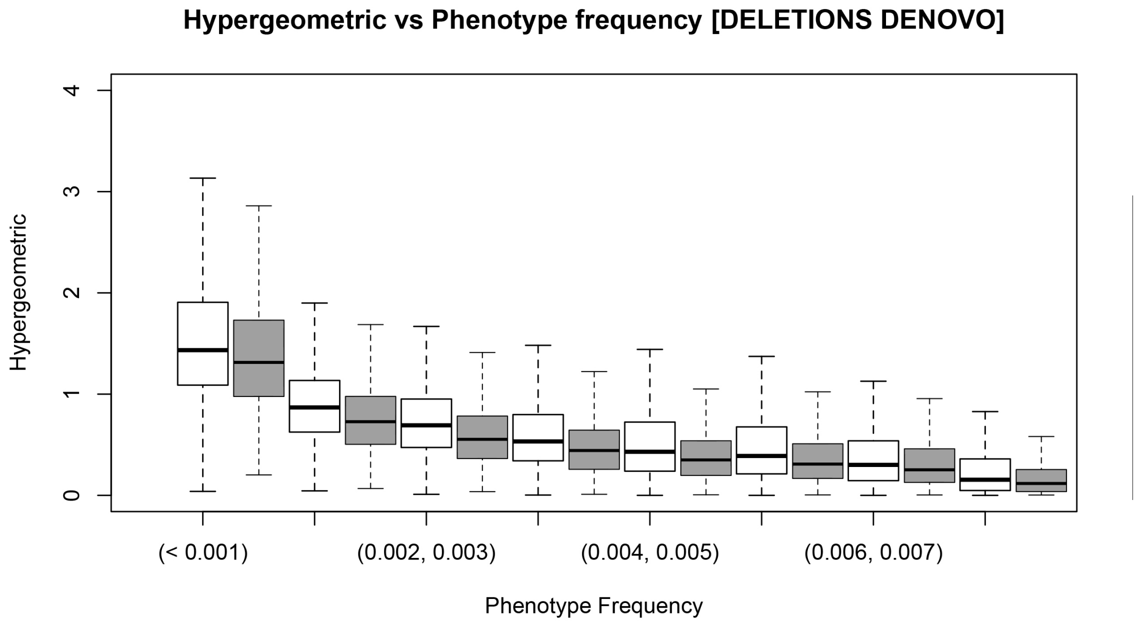

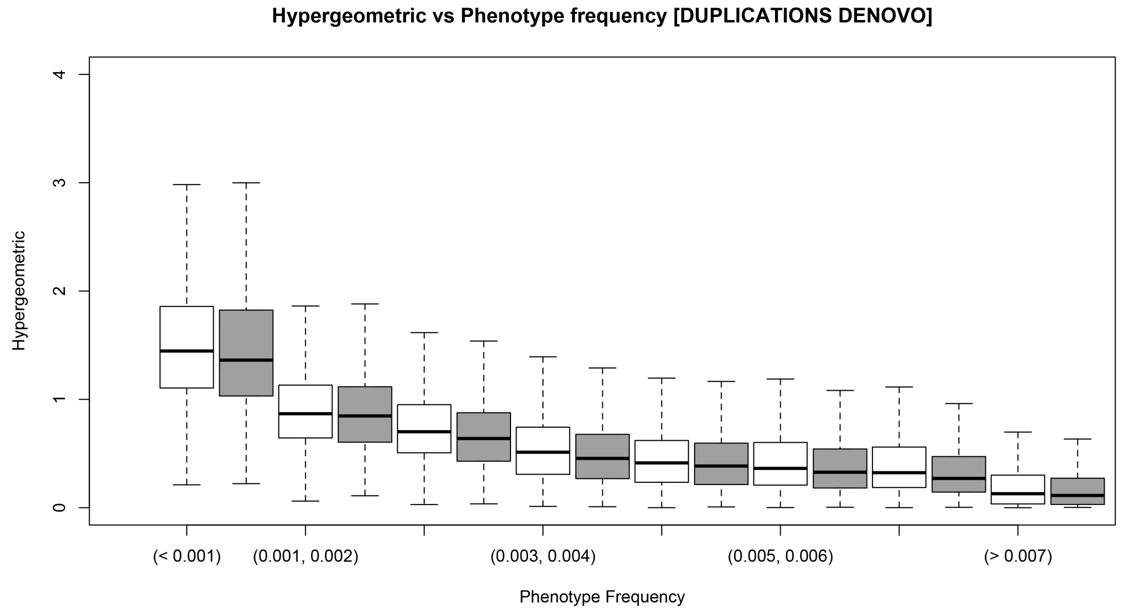

Figure S4. *Distribution of all the Hypergeometric Index values of the networks (between a phenotype and a locus), divided into different bins according to the frequency of the associated HPO term in the network: de novo deletions (upper plot) and de novo duplications (lower plot). Grey boxes represent the HyI distribution using randomly permuted nodes as null hypothesis and white boxes the HyI distribution using the intrinsic HyI null model. The high similarity of both HyI distributions confirms that the null hypothesis integrated in the HyI formulae is as expected.*

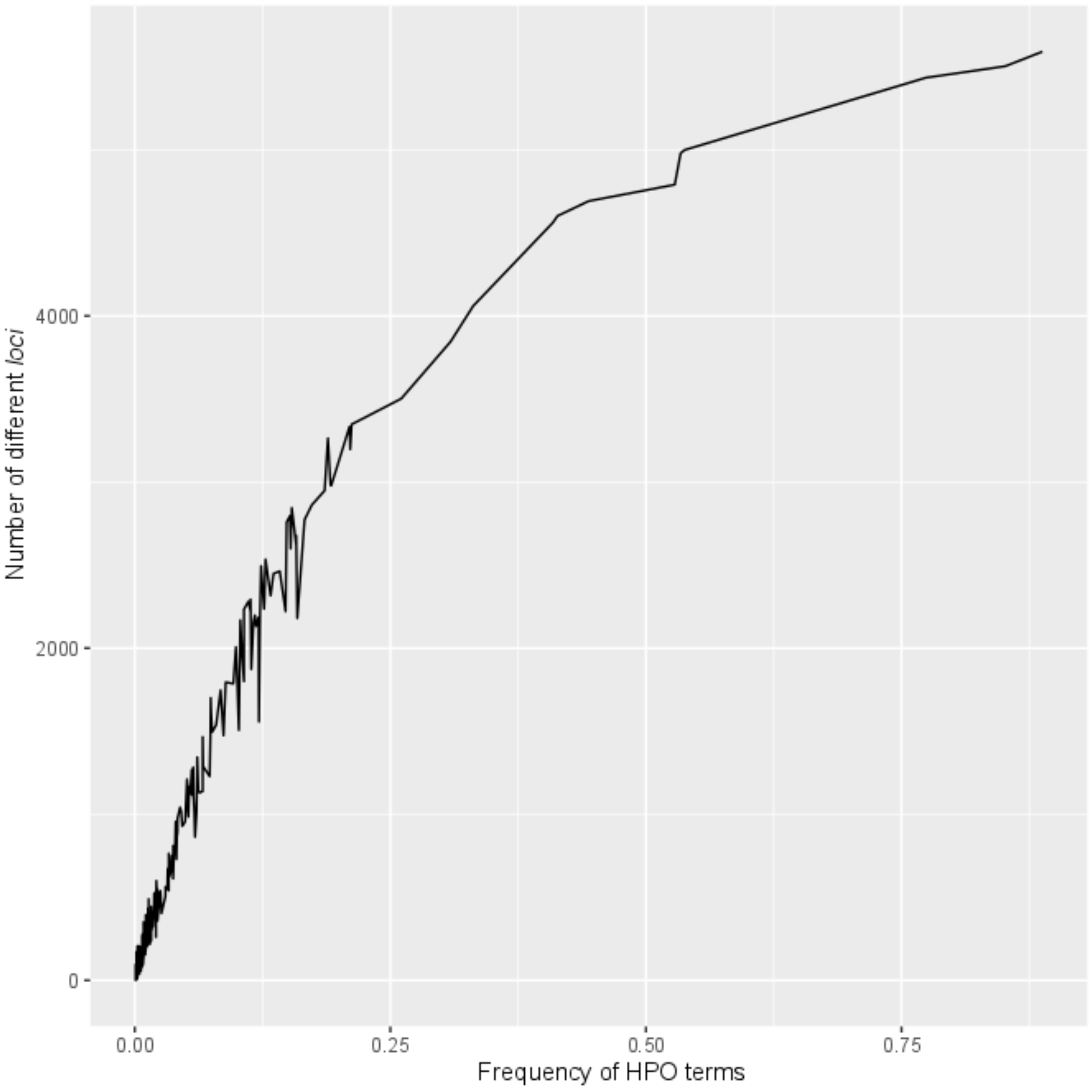

Figure S5. *HPO phenotypes frequency vs. number of associated loci. Phenotypes have been selected if their associated Hypergeometric value was higher than 0.*

Patients per *locus*, associated *loci* and HyI values.

The vast majority of patients are annotated with just one single CNV, therefore we can consider the number of patients and the number of CNVs like practically identical variables. We have performed two analyses in order to study the influence in the HyI of the number of patients/CNVs overlapping in the same *locus*. In the first analysis we studied the relationship between *loci* and patients/CNVs. We plotted the number of *loci* in function of the number of patients per *locus* (see Fig. S6). Although it is observed a small peak around 60 patients per *locus* that correspond to an unusual high accumulation of patients around some concrete regions in chromosomes 22 and 16, the mode of the remaining main distribution is about 10 patients per *locus*. This phenomenon can be explained by the fact that DECIPHER is composed by patients with rare genomic variants making it unlikely the big accumulation of patients with CNVs

overlapping on the same *locus*. In our second analysis we studied the effect of the number of patients per *locus* on the HyI values (see Fig. S7). This plot shows that the distributions of the HyI values are kept almost constant throughout all the values of the number of patients per *locus*, indicating a lack of correlation between a higher number of patients per *locus* with a higher HyI association values.

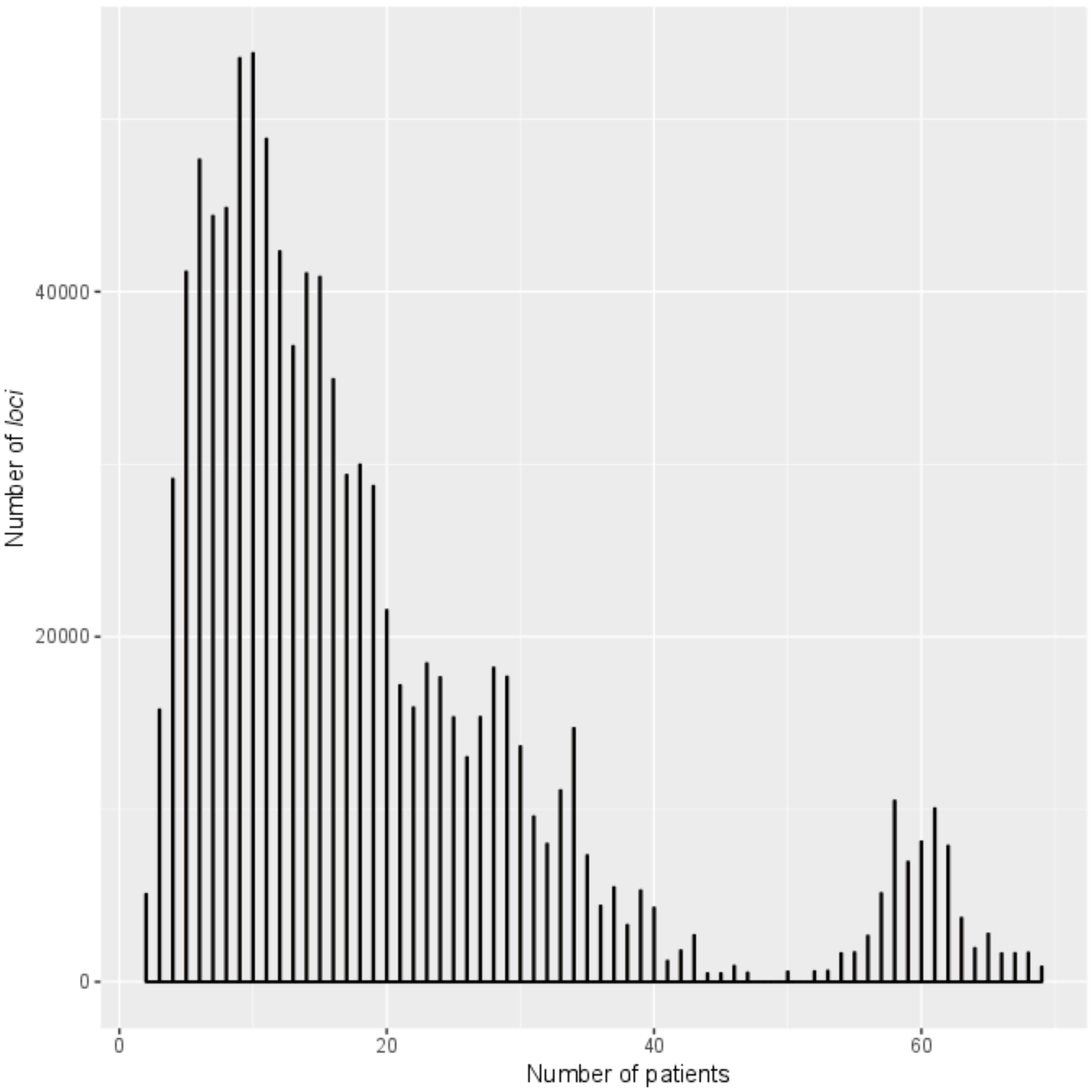

Figure S6. *Distribution of the number of loci shared by a determined number of patients.*

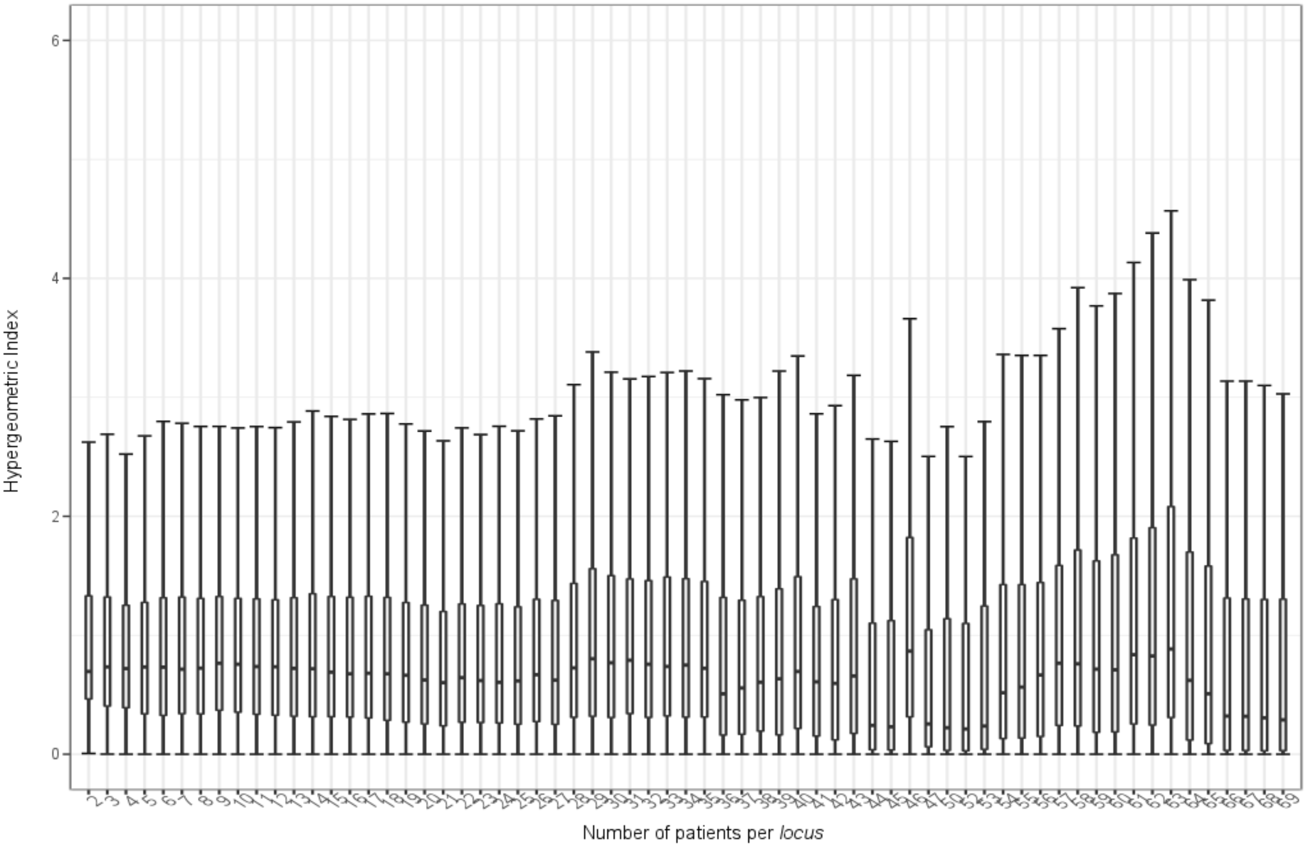

***Figure S7**. Distribution of Hypergeometric Index values (HyI) vs. number of patients per locus .*

These results indicate that HPO prevalence has influence (correlation) on HyI while the number of patients/CNVs per *locus* doesn't. This fact could be explained by the particular features of the DECIPHER tripartite network topology, with a group of prevalent HPOs connecting many patients and a mode of 10 patients connecting *loci*.

SECTION 4. Detailed study of a prevalent phenotype: "Abnormality of limb bone morphology".

We have studied in more detail the prevalent phenotype: "Abnormality of limb bone morphology" reported in Table 1 of the main manuscript. This is a prevalent phenotype in the DECIPHER dataset, with a frequency of 0.21. We have plotted the distribution of HyI values for all *loci*-HPO Terms associations for this phenotype (see Fig. S8). We observe that "Abnormality of limb bone morphology" (HP:0002813) is associated to 2 925 *loci*. The average, mode and range of the *loci*-HyI values distribution for this phenotype fit well into the general HyI distribution observed for other phenotypes in the same frequency range (see phenotype frequencies > 0.007 in Fig S4, Section 3). Around 99% of all the HyI values associated to this phenotype are below 2.0, the threshold used in this work for HyI values. The results obtained with this particular phenotype conform to the general trend observed for prevalent phenotypes associated to many *loci* with low HyI values, as we already have discussed in Section 3.

Although this phenotype shows a low range of HyI values, some of its *loci*-associations are significant with HyI values greater than 4.0. The *locus* associated to this phenotype with the maximum HyI value (around 5.0) is located on chromosome 7 and reported in Table 1 of the main manuscript. CNVs of 9 patients overlap in this *locus*, all of them diagnosed with "Abnormality of limb bone morphology", event that is pondered as very unlikely to be produced by chance (HyI null hypothesis) considering all the phenotype/patients/CNVs associations in the DECIPHER network. It has been shown that more specific (less prevalent) phenotypes tend to show higher HyI values, but it does not mean that more prevalent phenotypes cannot show a significant association index in a given *locus* if many patients share variant and phenotype.

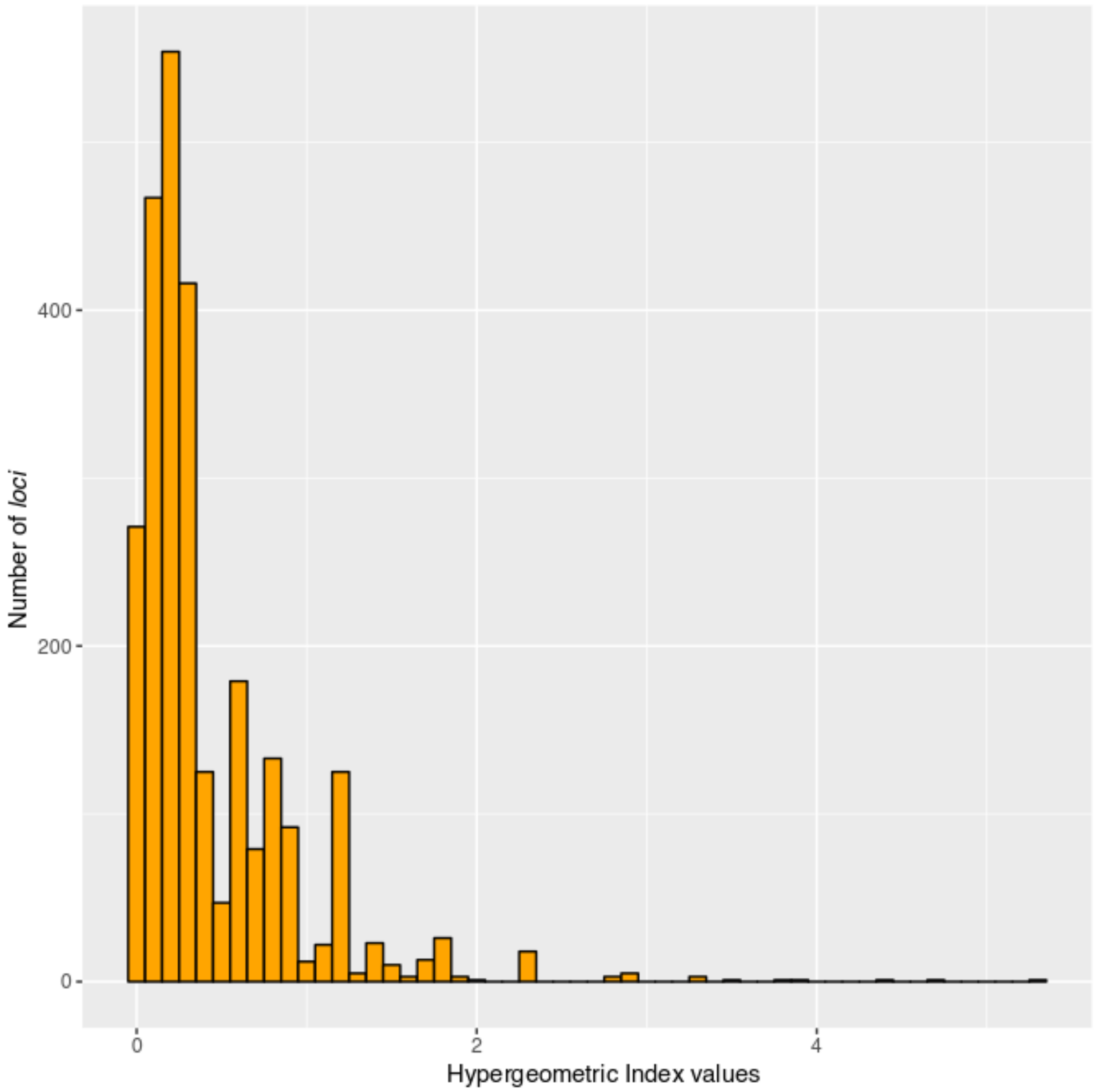

Figure S8. *Number of loci by Hypergeometric Index (HyI) values for the HPO term "Abnormality of limb bone morphology" (HP:0002813).*

SECTION 5. Retrospective clinical analysis of patients with microdeletions in 19p13.3

In the novel microdeletion syndrome described in Nevado et al. (6), our tool predicted three further clinical findings (not detected initially by clinicians): "abnormality of the kidney", "abnormality of the penis" and "abnormality of the connective tissue" with HyI ≥ 2.0 in 6 out of 13 patients (46%) (Table 4). Since the publication of this syndrome, a total of 38 patients have been identified with 19p13.3 microdeletions. In a retrospective review of these 38 patients, vesico-ureteral reflux (abnormality of the kidney) was found in 8 patients and other renal alterations were observed in 2 more cases. In addition, subtle hypospadias (abnormality of the penis) was observed in 1 of them and anomalies of clitoris or testicles were observed in 7 more cases. No clear signs of abnormality of the connective tissue were identified.

In summary, the 38 reanalyzed patients had the following phenotypes' penetrance values: renal anomalies 10/38 (26.31%); anomalies of the sexual organs 8/38 (21.05%); and no known cases of abnormality of the connective tissue. These results support the utility of this tool in assisting with clinical diagnosis.
